# Pharmacological targeting of CBP/p300 drives a redox/autophagy axis leading to senescence-induced growth arrest in non-small cell lung cancer cells

**DOI:** 10.1038/s41417-022-00524-8

**Published:** 2022-09-18

**Authors:** Mohammad Salik Zeya Ansari, Venturina Stagni, Angela Iuzzolino, Dante Rotili, Antonello Mai, Donatella Del Bufalo, Patrizia Lavia, Francesca Degrassi, Daniela Trisciuoglio

**Affiliations:** 1 Institute of Molecular Biology and Pathology, National Research Council of Italy (CNR), c/o Department of Biology and Biotechnology “C Darwin”, Sapienza University of Rome, Rome, Italy; 2 Laboratory of Cell Signaling, Istituto di Ricovero e Cura a Carattere Scientifico (IRCCS) Fondazione Santa Lucia, Rome, Italy; 3 Department of Drug Chemistry and Technologies, Sapienza University of Rome, Rome, Italy; 4 Preclinical Models and New Therapeutic Agents Unit, IRCCS-Regina Elena National Cancer Institute, Rome, Italy

**Keywords:** Cell biology, Lung cancer

## Abstract

p300/CBP histone acetyltransferases (HAT) are critical transcription coactivators involved in multiple cellular activities. They act at multiple levels in non-small cell lung carcinoma (NSCLC) and appear, therefore, as promising druggable targets. Herein, we investigated the biological effects of A-485, the first selective (potent) drug-like HAT catalytic inhibitor of p300/CBP, in human NSCLC cell lines. A-485 treatment specifically reduced p300/CBP-mediated histone acetylation marks and caused growth arrest of lung cancer cells via activation of the autophagic pathway. Indeed, A-485 growth-arrested cells displayed phenotypic markers of cell senescence and failed to form colonies. Notably, disruption of autophagy by genetic and pharmacological approaches triggered apoptotic cell death. Mechanistically, A-485-induced senescence occurred through the accumulation of reactive oxygen species (ROS), which in turn resulted in DNA damage and activation of the autophagic pathway. Interestingly, ROS scavengers were able to revert senescence phenotype and restore cell viability, suggesting that ROS production had a key role in upstream events leading to growth arrest commitment. Altogether, our data provide new insights into the biological effects of the A-485 and uncover the importance of the autophagic/apoptotic response to design a new combinatorial anticancer strategy.

## Introduction

Non-small cell lung cancer (NSCLC) represents about 85% of all lung cancers. In early-stage, NSCLC main treatment is surgical resection [1]. For advanced NSCLC, multimodal therapy is usually adopted to help shrink or, in some cases, completely remove the tumor [2]. Despite this, the rate of death is still high, and it is imperative to identify novel therapeutic targets to find new and effective treatment options.

In the last decade, histone modifications and chromatin modifiers have been shown to play key roles in cancer development, as they are involved in the regulation of gene expression and maintenance of genomic stability [3–5]. In this context, abnormal histone modification patterns have been reported in NSCLC, suggesting that changes in “histone code” may play a critical role in lung carcinogenesis and be predictive of the prognosis [6]. Additionally, it has been observed that altered expression and/or activity of histone-modifying enzymes may also contribute to lung cancer carcinogenesis [7, 8]. In particular, a variety of reports have implicated histone acetyltransferases (HAT) as both oncogenes and tumor suppressors [9].

p300 and CREB-binding protein (CBP) are members of the histone acetyltransferase family of transcriptional coactivators [9]. They mainly work by catalyzing lysine 18 and 27 histone H3 acetylation, but they also indirectly act by modulating some genes involved in DNA repair, cell growth, and angiogenesis by acetylating or interacting with different transcription factors [10**–**13].

In NSCLC, CBP and p300 genes are altered in about 10% of primary lung tumors and their aberrant expression seems to be associated with poor prognosis [14]. Recently, a whole genome sequencing study of normal lung tissue, primary tumors, and the corresponding metastases from NSCLC patients demonstrated that the mutation R397Q in p300 is highly detectable in the metastasis, indicating a potential role of p300 mutations in the metastatic spread of lung tumors [15]. Recent genome-wide sequencing studies revealed that ~10–15% of small cell lung cancers, another subset of lung cancer, harbor a gene truncation in the p300 or CBP, as well as loss-of-function mutations clustered in the HAT domain [16]. In line with these studies, p300 and CBP overexpression is regarded as an indicator of poor prognosis for lung cancer patients [14, 17–19]. Indeed, in lung cancer cells, p300 and CBP promote cell survival, migration, and invasion [18, 20], and p300 acetylates the transcription factor Snail, thus modulating E-cadherin expression and epithelial-mesenchymal transition (EMT) [17]. Recent studies show that the inhibition of p300/CBP by several small molecule inhibitors decreases cell proliferation and improves sensitivity to chemotherapy in lung cancer cells, suggesting a relevant role of p300 and CBP in this tumor histotype [20**–**24]. Thus, p300/CBP appears to be a promising druggable target in view of the global reach that one would achieve in therapeutic terms.

Recently, the spiro oxazolidinedione compound (indicated as A-485) has been reported as the first drug-like potent and selective catalytic inhibitor of p300/CBP, targeting the HAT domain. This compound exhibits potent in vitro inhibitory activity (at nanomolar concentrations), low clearance, and high oral bioavailability, and it selectively affects cancer cell viability in vitro and in vivo [25**–**27], with initial promising indications that it may also be effective in NSCLC [21]. In this study, we have thoroughly evaluated the mechanism of action of A-485 in model NSCLC cell lines, dissecting its effect on cell death pathways, particularly with respect to the modulation of autophagy, senescence, and their crosstalk. We showed that A-485 reduces NSCLC cell growth and drives tumor cells to undergo senescence through the activation of oxidative stress and autophagy.

## Results

### A-485 induces cell growth arrest without activation of apoptotic cell death

To investigate whether A-485 might be effective in NSCLC cells, we firstly exposed A549 cells (Fig. S1A–D) and H1299 (Fig. S1E–H) cells to A-485 in a time course experiment and tested its effect on histone acetylation marks by Western immunoblot analysis. As expected, A-485 effectively reduced levels of Ac-H3K18 and Ac-H3K27, primary targets of p300/CBP acetylation activity, in a dose- and time-dependent manner in both cell lines (Fig. S1).

**Fig. 1:**
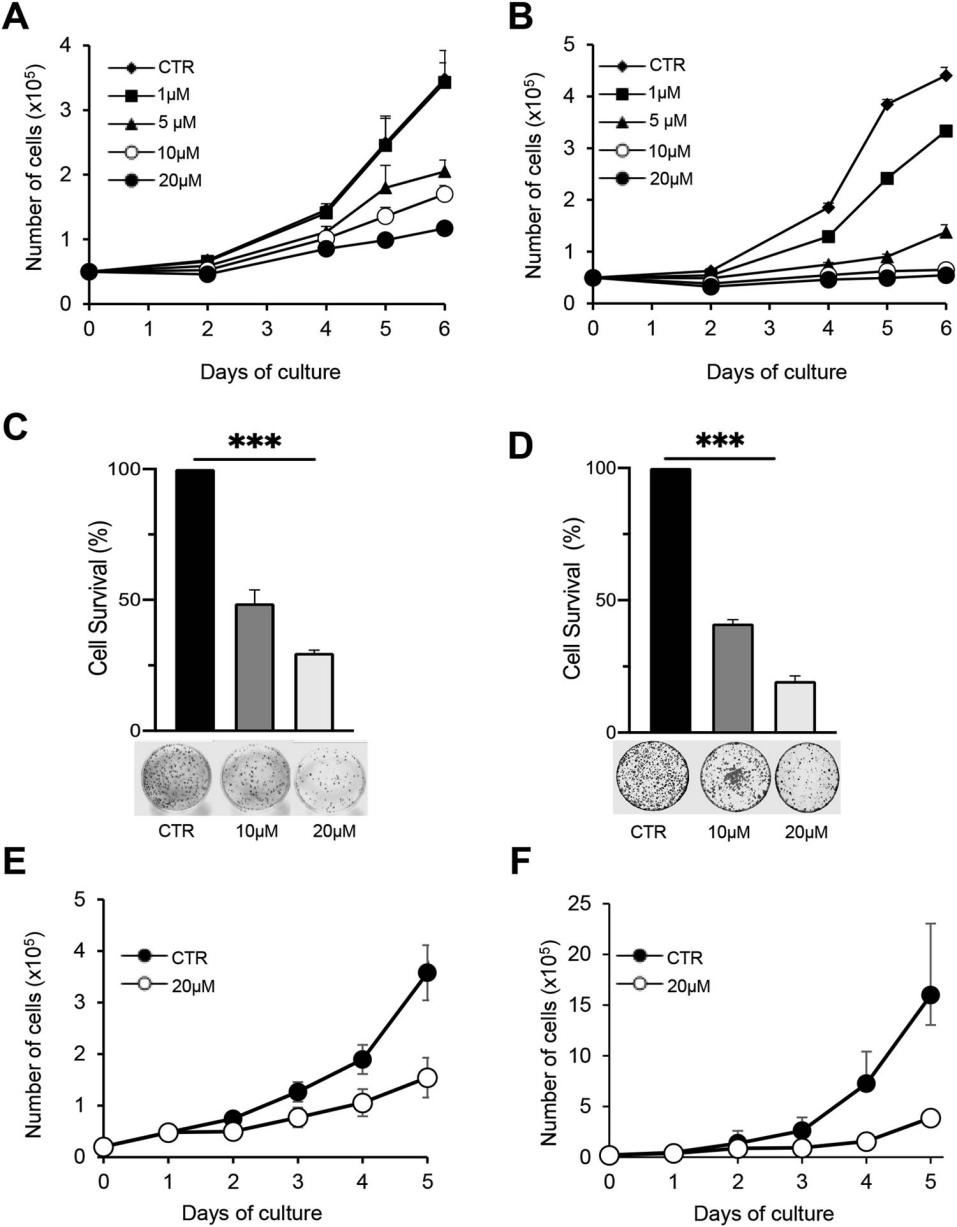
A-485 inhibits cell growth in NSCLC cell lines. Cell growth curves of A549 (**A**) and H1299 (**B**) cell lines, untreated or treated with A-485 at the indicated doses. About 5 × 10^4^ cells were seeded on day 0 and treated on day 1. Clonogenic assay of A549 (**C**) and H1299 (**D**) cell lines, untreated or treated for 72 h with A-485 at the indicated doses. Representative images of colonies of A549 and H1299 cells stained with crystal violet are shown. The histograms represent the % of colony-forming cells relative to untreated ones. The results were statistically evaluated using Tukey’s multiple comparisons test, ***p < 0.0001. Cell growth curves of H460 (**E**) and H1650 (**F**) cell lines, untreated or treated with A-485 at the indicated dose. About 5 × 10^4^ cells were seeded on day 0 and treated on day 1. **A**–**F** CTR represents 0.1% DMSO treated cells. The graphs represent the mean ± SD of three independent experiments.

Having corroborated the inhibitory effect of A-485 on histone acetylation, we evaluated the A-485 effect on cell proliferation and survival (Fig. 1).

A-485 led to a decrease in cell proliferation in a dose-dependent manner as compared with control cells, starting from day 4 of growth (72 h after treatment) in both A549 (Fig. 1A) and H1299 (Fig. 1B) cell lines. More importantly, A-485 also led to an impairment of the colony-forming ability in both analyzed cell lines in a dose-dependent fashion. Indeed, A549 cells exhibited about 50 and 75% reduction in colony formation at 10 and 20 µM of A-485, respectively (Fig. 1C), while H1299 cells showed about an 80% decrease at the highest tested dose (Fig. 1D). Moreover, A-485-induced cell growth inhibition was of general application because it was observed also in other NSCLC cell lines e.g., H460 (Fig. 1E) and H1650 (Fig. 1F) cells.

**Fig. 2:**
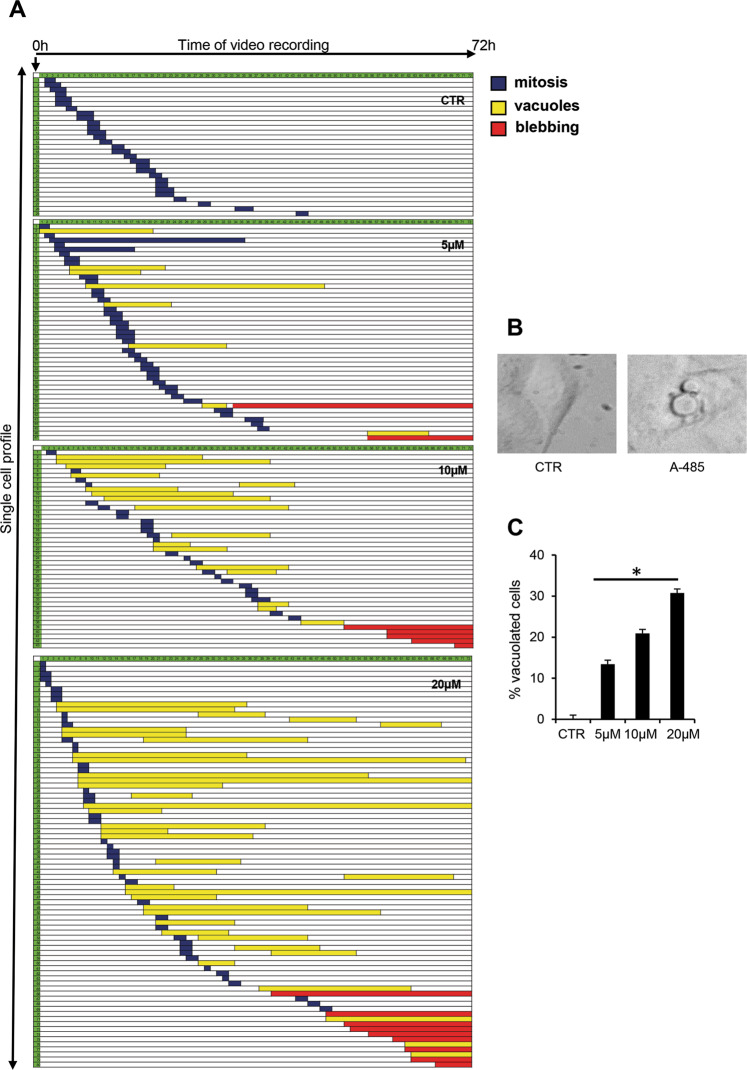
Single-cell time-lapse analysis identifies morphological changes induced by the A-485 treatment. **A** The panels represent the fate of individual cells in samples untreated or treated with A-485 at the indicated doses. Each line depicts a single cell recorded over time that shows a specific phenotype. Cells are arranged according to the timing of morphological changes; the length of each line indicates the duration of cell division (blue), vacuolation (yellow), or the appearance of apoptotic blebbing (red). Recorded cells: 0.1% DMSO treated cells (CTR) = 84; 5 μM = 116; 10 μM = 103; 20 μM = 148, two independent experiments. **B** Single snapshots from video-recorded A549 cells, untreated or treated with A-485. **C** The histogram represents the percentage of cells presenting vacuolation under increasing A-485 concentrations. Mean ± S.D were statistically evaluated using Tukey’s multiple comparisons test, *p < 0.05.

To explore the fate of cells in response to A-485 and to follow dynamically the cellular response of treated cells, we recorded A549 cells by phase contrast time-lapse microscopy from the start of A-485 treatment over the next 72 h. Single-cell analysis was carried out to identify the fate of every single video-recorded cell (Fig. 2). The video-recording data revealed a dose-dependent change in the dynamic phenotypes of treated cells in terms of timing of mitotic entry as well as appearance and persistence of cytoplasmic vacuoles. As shown in Fig. 2A, control cells progressed from whichever cell stage they were at recording onset up to the onset of mitosis, which they completed in about 2 h. A-485-treated cells were generally delayed in mitotic entry compared to controls, indicating they were stalled in interphase. No signs of mitotic abnormalities were observed in both control and A-485 treated ones. Phenotypic changes in cell morphology were noted in treated samples, including an enlarged and flattened phenotype and the appearance of large vacuoles (Fig. 2B). The highest A-485 dose remarkably increased the cell population with large vacuoles (Fig. 2C), yet only a small percentage of these cells underwent apoptotic blebbing after vacuole appearance. Moreover, few cells showed the apoptosis phenotype, suggesting that A-485 alone cannot induce apoptosis. This data is consistent with our cytofluorimetric experiments (Fig. S2A). Indeed, A-485 arrests cells in the G0/G1 phase of the cell cycle for 72 h, and this arrest persists without no signs of DNA fragmentation, indicative of apoptosis. Even after 120 h of treatment, cultures did not show any accumulation of the sub-G1 peak population (Fig. S2B). Collectively, these results indicate that A-485 causes a growth arrest of NSCLC cells that is associated with phenotypic changes in cell morphology without activation of the apoptotic program.

### A-485 induces a canonical autophagic pathway that prevents cell death

As cytoplasmic vacuolization is a common mark of autophagy activation [28], and acetylation acts at multiple levels in the autophagic process [29], we tested the hypothesis that inhibiting p300/CBP by A-485 might act through activating autophagy. Thus, we analysed the microtubule-associated protein 1 A/1B-light chain 3 (LC3), which plays essential role during autophagy, acting in both substrate selection and autophagosome formation [30].

**Fig. 3:**
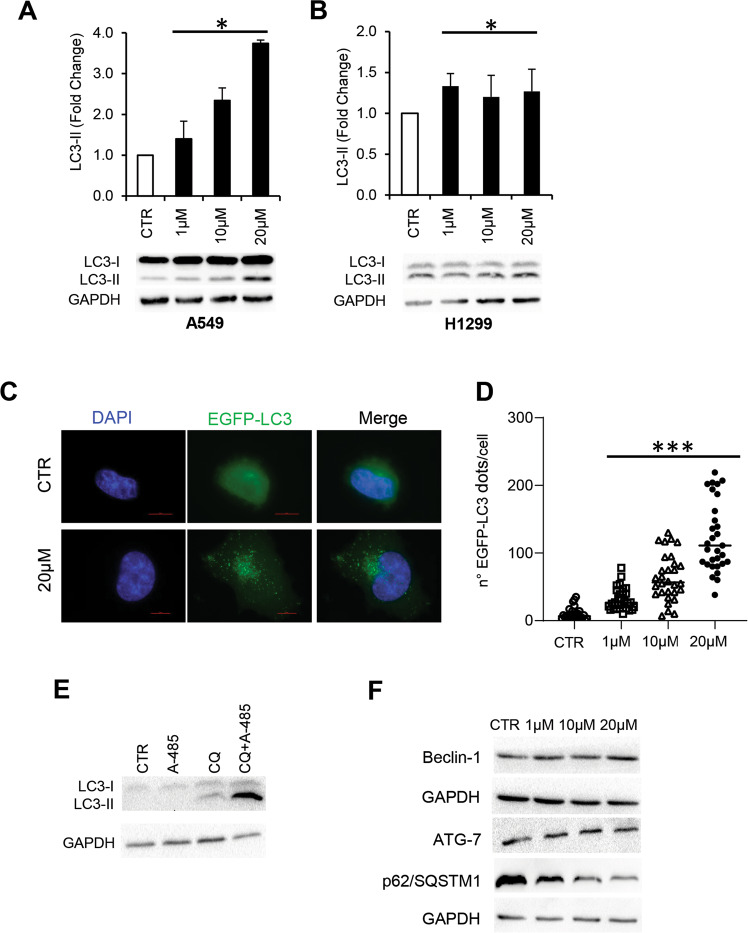
A-485 induces autophagy in NSCLC cells. Western blot analysis of LC3BI-II protein level in A549 (**A**) and H1299 (**B**) cells, untreated or treated with the indicated doses of A-485 for 24 h. LC3B-II levels were quantified by densitometric analyses using ImageLab software and the relative levels of proteins were expressed in the histograms as fold changes of treated versus untreated samples after GAPDH normalization. Results represent the mean ± SD of three independent experiments. Mean ± S.D were statistically evaluated using Tukey’s multiple comparisons test, *p *<* 0.05 **C** Representative images of autophagosomal structures by fluorescence microscopy in H1299 cells stably transfected with EGFP-LC3B vector (H1299/EGFP-LC3) and treated with A-485 for 24 h, and **D** Quantification of EGFP-LC3 dots/cell. The results were statistically evaluated using 2way ANOVA followed by Dunn’s multiple comparison test, ***p *<* 0.001*.* **E** Western blot analysis of LC3B I-II protein level in A549 cells treated with A-485 (20 μM), the late-stage inhibitor Chloroquine (CQ, 25 μM) alone or in combination for 24 h. **F** Western blot analysis of Beclin-1, ATG7, p62/SQSTM1 protein levels in A549 cells, untreated or treated with the indicated doses of A-485 for 24 h. Western blots representative of two independent experiments with similar results are shown. GAPDH is shown as a loading control. CTR represents 0.1% DMSO treated cells.

As reported in Fig. 3A, B, A-485, already at 1 μM, increases the conversion of the non-lipidated (LC3-I) to the conjugated (LC3-II) LC3 form, in both A549 and H1299 cells, notwithstanding the differential abundance of the two forms in these cell lines at steady state. As LC3-II is a typical autophagosome marker, these data indicate that A-485 up-regulates autophagosome formation in lung cancer cells. To confirm these data, H1299 cells stably expressing EGFP-LC3 (H1299-EGFP-LC3) were exposed to increasing doses of A-485 for 24 h and observed by fluorescence microscopy. As shown in Fig. 3C, the green fluorescence of EGFP-LC3 is uniformly spread throughout the cell in untreated cells. On the contrary, treatment with A-485 induces a dose-dependent redistribution of the LC3 protein in the cytoplasm with the formation of autophagosomes appearing as fluorescent spots. EGFP-LC3 dots per cell were significantly increased in response to A-485 (Fig. 3D). To discriminate between an A-485-dependent increase in autophagosome formation or blockade of maturation, we used the late-stage lysosomal inhibitor, CQ [31]. The increasing accumulation of LC3-II in A549 cells exposed to the combination of A-485 and CQ compared to a single treatment, demonstrated that A-485 induced a complete autophagic flux (Fig. 3E). To extend these observations, we further analysed A549 cells for the expression of the autophagy markers Beclin-1 and Atg7, implicated in autophagosome nucleation and elongation, and the protein cargo p62/SQSTM1. We found a slight increase in Atg7 and Beclin-1 levels and a concomitant reduction of p62/SQSTM1 levels when compared to the control (Fig. 3F), strengthening the conclusion that A-485 inhibition induces a canonical autophagic pathway.

**Fig. 4:**
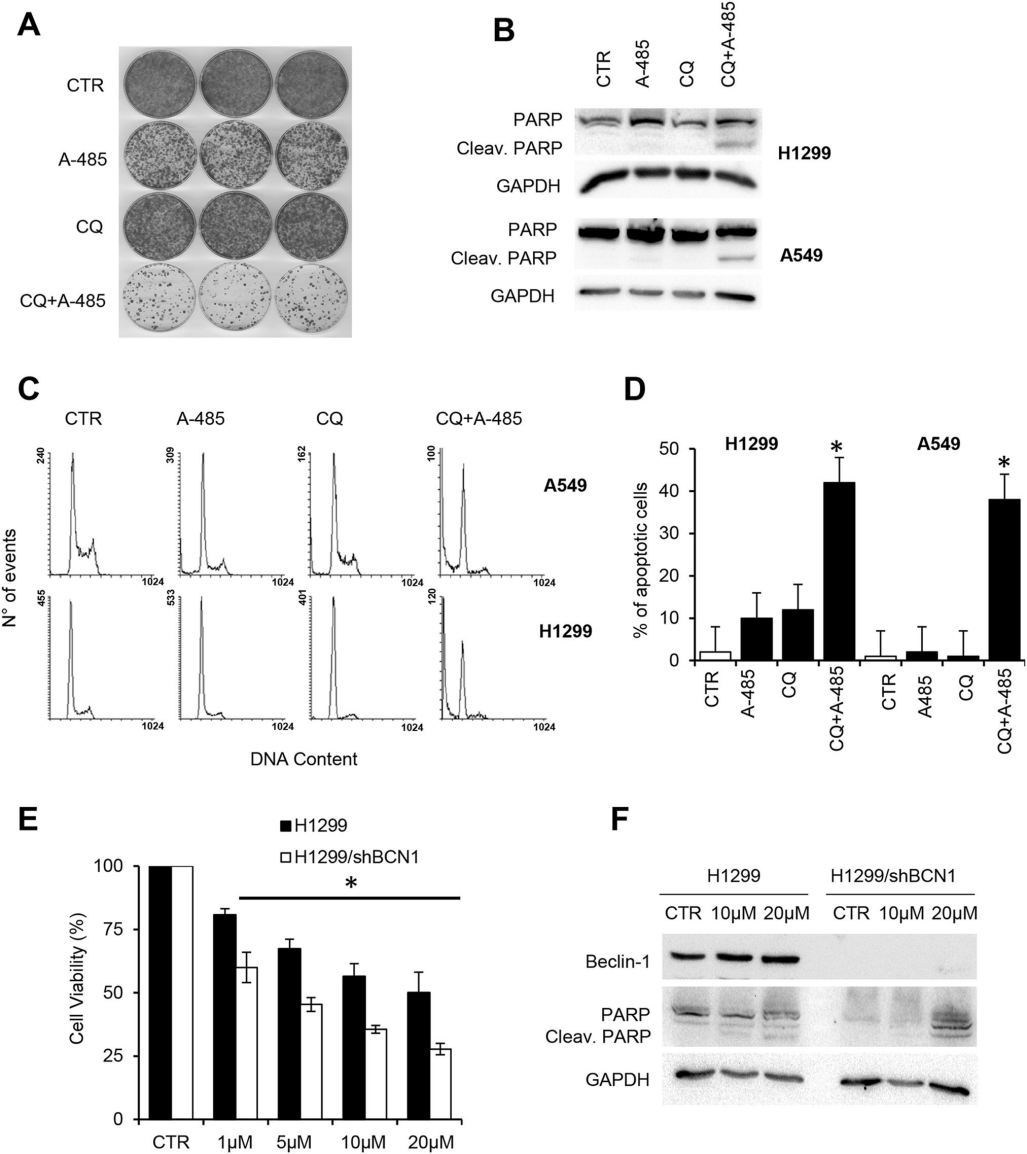
Pharmacological or genetic autophagy blockade induces cell death upon A-485 treatment. **A** Clonogenic assay of A549 cells, untreated or treated with A-485 (10 μM), the late-stage inhibitor Chloroquine (CQ, 25 μM) alone or in combination for 72 h. Representative images of cell colonies stained with crystal violet are shown. **B** Western blot analysis of PARP cleavage in H1299 and A549 cells, untreated or treated with A-485 (10 μM) and CQ (25 μM) alone or in combination for 72 h. **C** Representative flow cytometric analysis of the sub-G1 fraction and relative quantification (**D**) in A549 and H1299 cells, untreated or treated for 72 h with 10 μM of A-485, 25 μM of CQ, or the combination of A-485 and CQ. The histogram represents the % of apoptotic cells. The asterisk indicates *p* < 0.05 as compared with untreated cells (*t*-test). **E** MTT analysis of H1299 cells and H1299 cells stably silenced for the expression of Beclin-1 (H1299/shBCN1). Cells were incubated with increasing concentrations of A-485 for 72 h. The results are reported as the “viability of drug-treated cells/viability of control cells” × 100. Means ± SD from three different experiments are shown. The results were statistically evaluated using two-way ANOVA followed by Sidak’s multiple comparisons test,*p *<* 0.05. **F** Western Blot analysis of cleaved PARP and Beclin-1 in H1299 cells and H1299/shBCN1 cells treated with A-485 at the indicated concentrations. **B**, **F** Western blots representative of two independent experiments with similar results are shown. GAPDH level was used as a loading control. **A**–**F** CTR represents 0.1% DMSO treated cells.

To analyse whether activation of the autophagic pathway was implicated in the cell survival response to A-485, we evaluated the biological outcome of autophagy inhibition by using CQ in combination with A-485. As shown in Fig. 4A, A-485 induced a significant fall in colony formation compared to control cells, while CQ treatment alone only marginally affected the clonogenic capacity of A549 cells. Notably, a reduction in the number of colonies was observed after A-485 and CQ cotreatment compared to cells exposed to A-485, thus indicating that inhibition of autophagy increases cell death induced by the A-485 treatment.

Next, the effect of single or combined treatments with A-485 and CQ on H1299 and A549 cell death was analysed by assessing the cleavage of Poly(ADP-ribose) polymerase (PARP), a proteolytic substrate of caspases. As expected, A-485 alone did not activate caspase-mediated apoptosis, while the cleavage of PARP was well evident only after cotreatment of A-485/CQ, in both cell lines analysed (Fig. 4B). Concordantly, flow cytometry analysis of the sub-G1 peak (indicative of apoptotic cell death) showed that the percentage of apoptotic cells was significantly increased in the combination group compared to the single treatments in both cell lines (Fig. 4C, D). These data support autophagy as the mechanism of A-485-mediated cytoprotection in lung cancer cell lines.

To corroborate the finding that targeting the autophagic pathway could sensitize NSCLC cells to A-485, we tested the effect of A-485 on H1299 cells silenced for the expression of Beclin-1, a protein acting at the early steps of autophagy. Upon A-485 treatment, selective knockdown of Beclin-1 resulted in a decreased percentage of viable cells at all tested doses (Fig. 4E) and in the induction of cleaved PARP (Fig. 4F) when compared to control-transfected H1299 cells. Taken together, these findings strongly indicate that following A-485 treatment, the inhibition of autophagy results in apoptosis induction and overall loss of cell viability.

### A-485 induces senescence phenotype in NSCLC cell lines

As A-485 leads to senescence in melanoma and breast cancer cells [27, 32], to further explore the mechanism through which A-485 exerts its antiproliferative effects in NSCLC cells, we evaluated whether A-485 induced senescence, a stable proliferation arrest evoked by a plethora of stress-inducing factors [33].

**Fig. 5:**
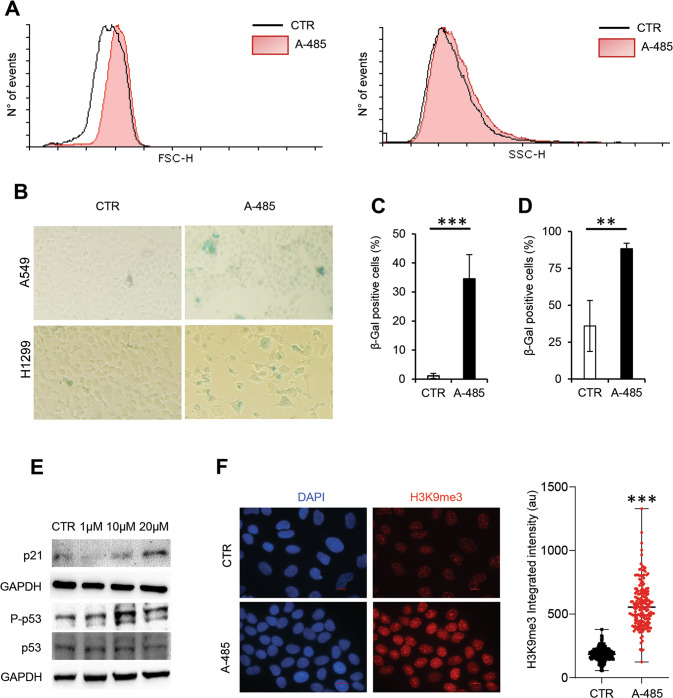
A-485 treatment induces cellular senescence in NSCLC cells. **A** The size and granularity of control and A-485 treated cells were determined by forward (FSC-H) and side scatter (SSC-H) flow cytometry analysis. Representative monoparametric analysis is shown. **B** Representative microscopic images of SA-β-gal positive (blue stained) senescent A549 and H1299 cells after 120 h of A-485 treatment (20 μM). **C**, **D** Quantification of SA-β-gal positive in A549 (**C**) and H1299 (**D**) cells. Data were means ± SD of three independent experiments; at least 100 cells from ten randomly chosen fields were counted in each experiment. The results were statistically evaluated using *t*-test **p < 0.001, ***p < 0.0001. **E** Western blot analysis of p21 expression, p53 phosphorylation, and expression in A549 cells after A-485 treatment (120 h, 20 μM). GAPDH is shown as a loading control. A western blot representative of two independent experiments with similar results is shown. **F** Immunofluorescence images of histone H3K9me3 abundance in A549 cells after A-485 treatment (120 h, 20 μM) (left panel) and relative quantification of histone signal intensity (right panel) based on immunofluorescences staining using H3K9me3 (red) antibody and nuclear counterstaining using DAPI (blue). 40x objective. Scale bar = 10 μm. The results were statistically evaluated using Mann–Whitney test, ***p < 0.0001. CTR represents 0.1% DMSO treated cells.

Senescent cells are defined by morphological alterations and characterized by increased SA-β-galactosidase activity. Thus, A549 cells were treated with A-485 and were subjected to cytofluorimetric measurements of the cell size and granularity, as well as a senescence-associated-β-galactosidase (SA-β-Gal) assay. Figure 5A shows a monoparametric analysis of forward scatter (FSC-H) and side scatter (SSC-H) intensity, representative of the cell size and internal complexity (i.e., granularity). Median FSC and SSC values were lower in untreated compared to treated cells, indicating that A-485 cultures contained a higher proportion of bigger cells compared to untreated cultures. To confirm these observations, we performed an SA-β-Gal assay in both H1299 and A549 cells, as depicted in Fig. 5B, C**:** an increased frequency of SA-β-Gal positive cells was observed after 120 h in A-485 treated samples compared with A549 (Fig. 5B, C) and H1299 (Fig. 5B, D) controls, suggesting the activation of a senescence phenotype. To corroborate our results, we also evaluated in A549 cells, harboring the p53^wt^ gene, the effect of A-485 on two further markers of the senescence program, i.e., the p53/p21 axis and histone H3 trimethylation at K9 (H3K9me3). As shown in Fig. 5E, F phosphorylation of p53 markedly increased, concomitant with increased abundance of both p21 and H3K9me3, upon 120 h A-485 treatment as compared to controls. Together, these results indicate that the A-485 treatment induced cellular senescence.

### Induction of ROS contributes to A-485-mediated senescence and autophagy

**Fig. 6:**
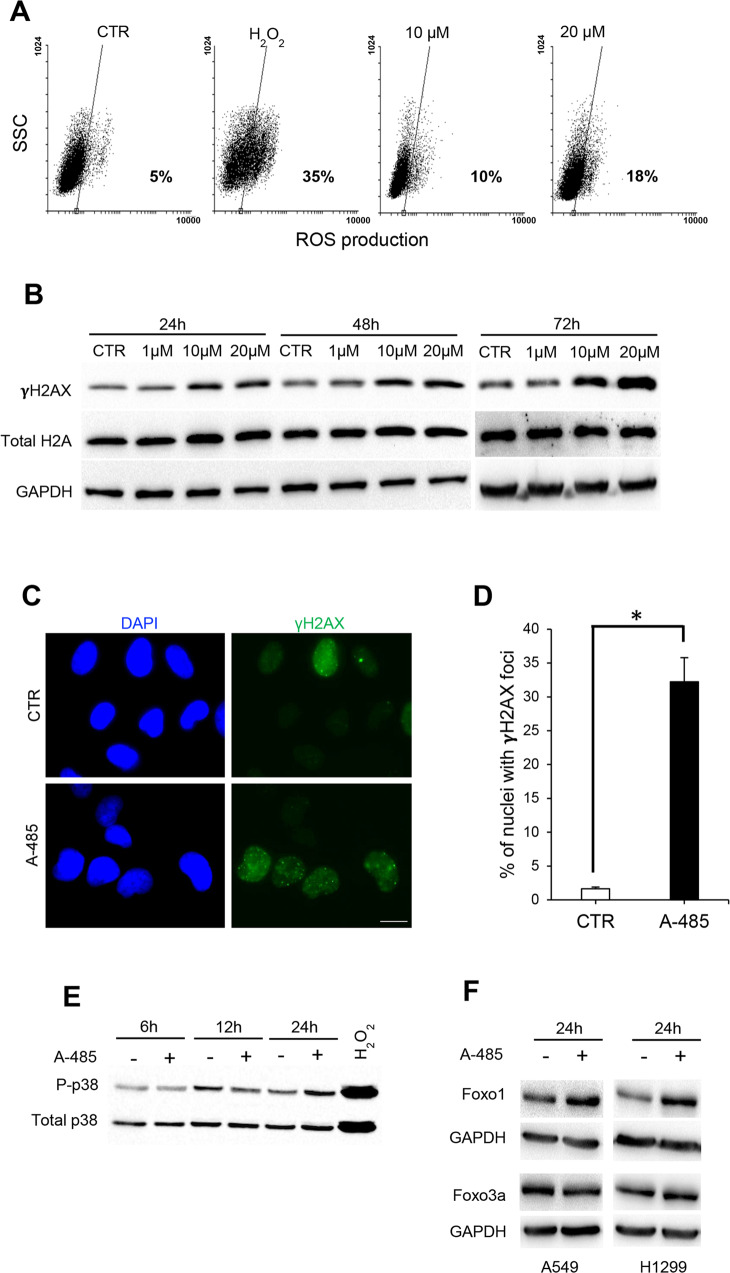
Induction of ROS contributes to A-485-mediated senescence. **A** Representative flow cytometric analysis of ROS levels by H_2_DCFDA assay in A549 cells untreated or treated with A-485 for 72 h. H_2_O_2_ was used as a positive control. The percentage of cells positive for H_2_DCFDA staining is shown. **B** Western blot of γH2AX and total H2A in A549 cells untreated or treated with different concentrations of A-485 for 24, 48, or 72 h. **C** Representative images of γH2AX nuclear foci by fluorescence microscopy in A549 untreated or treated with A-485 for 72 h, and **D** quantification of the percentage of cells showing γH2AX nuclear foci. The results were statistically evaluated using *t*-test, *p *<* 0.05. **E** Western Blot analyses of a phosphorylated and total form of p38 in A549 cells treated with A-485 (20 μM) for the indicated times. H_2_O_2_ was used as a positive control. **F** Western blot of Foxo1 and Foxo3a expression in A549 and H1299 cells, untreated or treated with A-485 (20 μM) for 24 h. GAPDH is shown as a loading control. Western blots representative of three independent experiments with similar results are shown. CTR represents 0.1% DMSO treated cells.

Given that several reports suggest a link between oxidative stress, autophagy, and senescence in cancer cells [34, 35], we analysed the impact of A-485 treatment on ROS production. As shown in Fig. 6A, A-485 treatment determines a dose-dependent intracellular ROS accumulation, reaching about 20% of ROS positive cells with the highest dose compared to a 5% baseline level.

The generation of ROS might lead to DNA damage, and both can be critical in maintaining senescence [36]. To assess whether A-485 induced DNA damage, we performed a western blot analysis of γH2AX, a histone variant specifically recruited to DNA breaks and readily phosphorylated in the early steps of the DNA damage response (DDR). Of note, in A-485-treated A549 cells, we found an increased level of γH2AX histone already evident after 24 h treatment and persisting until 72 h (Fig. 6B). We also scored the abundance of γH2AX nuclear formation by immunofluorescence in A549 following A-485 treatment. As depicted in Fig. 6C, D A-485 yields a significant increase in the percentage of cells showing γH2AX foci, thus confirming that A-485 induces DDR signaling.

**Fig. 7:**
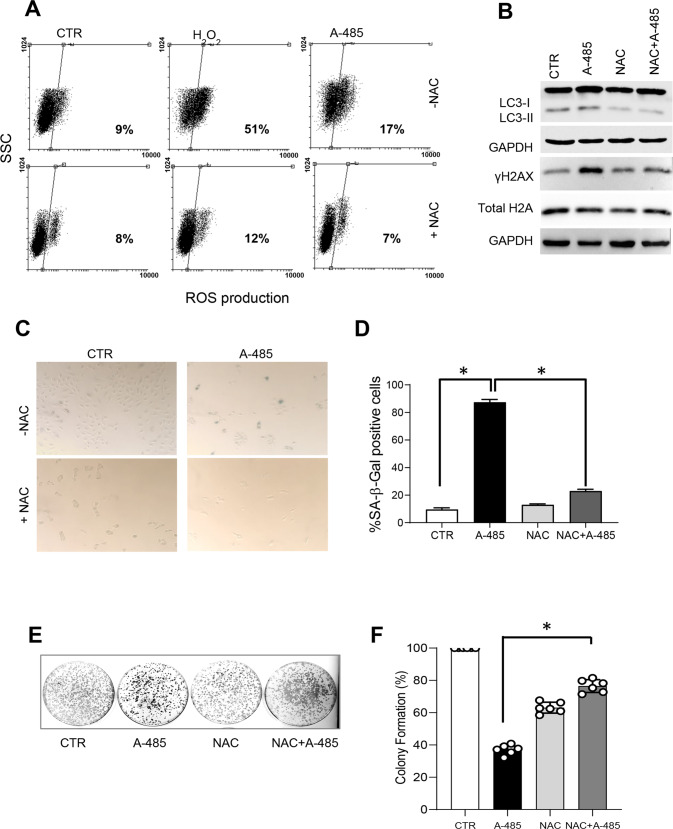
Inhibition of ROS prevents A-485-induced autophagy and senescence. **A** Representative flow cytometric analysis of ROS levels by H_2_DCFDA assay in A549 cells untreated or treated with A-485 for 72 h alone or in combination with NAC (10 mM) for 72 h. H_2_O_2_ was used as a positive control. The percentage of cells positive for H_2_DCFDA staining is shown. **B** Western blot of LC3B-II and γH2AX protein levels in A549 cells incubated with A-485 (20 μM) alone or in combination with NAC (10 mM) for 72 h GAPDH is shown as a loading control. Western blots representative of three independent experiments with similar results are shown. **C** Representative microscopic images of SA-β-gal positive (blue stained) senescent cells and **D** relative quantification of β-galactosidase-positive A549 cells incubated with A-485 (20 μM) alone or in combination with NAC (10 mM) for 120 h. Data were means ± SD of three independent experiments; at least 100 cells from ten randomly chosen fields were counted in each experiment. The results were statistically evaluated using *t*-test, *p *<* 0.05. **E** Representative images of colonies and **F** quantification of clonogenic assay of A549 cell line treated or not treated with A-485 (10 μM), with NAC (10 mM) alone or in combination for 72 h. CTR represents 0.1% DMSO treated cells. The results were statistically evaluated using *t*-test *p < 0.05.

Notably, in response to A-485 treatment, we also found activation of p38, one of the main stress-induced mitogen-activated protein kinases (MAPK) (Fig. 6E), and an increase in the ROS-responsive transcription factor Foxo1, but not in Foxo3a, another member of the forkhead family of transcription factors, in response to A-485 treatment (Fig. 6F). Because Foxo1—unlike Foxo3—is selectively implicated in the control of the redox balance, this finding raised the question of whether oxidative stress may be implicated in autophagy and cellular senescence observed in A-485 treated NSCLC lines. To answer this question, we used NAC, a commonly used ROS scavenger [37, 38]. First, we analysed the impact of NAC treatment on ROS production. As shown in Fig. 7A, A-485 treatment (72 h) determines ROS accumulation, that is completely abrogated in presence of NAC. Moreover, we compared γH2AX histone levels and LC3-I to LC3-II conversion with and without NAC to assess whether NAC modulated DNA damage and autophagy activation by A-485. As shown in Fig. 7B, the increase of γH2AX and LC3-II levels by A-485 was prevented in cells pretreated with NAC, indicating that A-485-induced DNA damage and autophagy were downstream events of ROS formation. We further tested A-485 effects on senescence after pretreatment with NAC. Notably, as reported in Fig. 7C, NAC resulted in a drastic reduction of A-485-induced senescent cells in the A549 cell line. These results indicate that ROS production is required for the establishment of cellular senescence in response to A-485.

To determine whether the inhibition of ROS formation was paralleled by a cytoprotective effect over A549 cells, we further tested the effect of NAC treatment on clonogenicity in response to A-485. As reported in Fig. 7C, when NAC was used in combination with A-485, the colony number increased in comparison with A549 cells treated with A-485 alone. In conclusion, a mutual regulation exists between oxidative stress, autophagy, and senescence in A549 cells exposed to A-485, which can be counteracted by the anti-oxidant NAC activity.

## Discussion

The role of p300/CBP in cancer has been described, yet targeting p300/CBP has proven challenging because only a few compounds showed HAT selectivity. In this study, we used A-485 as a strategy to assess the relevance of p300/CBP therapeutic inhibition in the NSCLC setting and to decipher how A-485 affects p300/CBP-dependent cellular death pathways.

We provide evidence that A-485 inhibits cancer cell proliferation in different NSCLC cell lines with no signs of apoptosis activation. Indeed, A-485 treated cells displayed phenotypic markers of cell senescence, including increased cell size, senescence-associated-β-galactosidase activity, increased H3K9me3, and upregulation of p21, which facilitate cell-cycle arrest [39, 40]. Our results converge with and extend previous data reporting a connection between p300/CBP and induction of cellular senescence: indeed, p300 was found to be a primary driver of the senescent phenotype in normal cells [41, 42] and p300/CBP inhibition by either C646 or A-485 HAT inhibitors led to senescence in melanoma and breast cancer cells [27, 32, 43]. In addition, genetic inhibition of CBP promoted cell differentiation and p53-dependent cell senescence in chronic myeloid leukemia independent of BCR-ABL status [44].

It is interesting to note, from a mechanistic point of view, that A-485 drove NSCLC cells to senescence independent of their genetic background. Indeed, A-485 induced senescence via a canonical p53/p21 axis in the A549 cell model (p16^-^, p53^+^, Rb^+^), yet also triggered the senescence pathway in H1299 cells that are defective in both the p53-p21 and p16-Rb axes. A recent paper has demonstrated that downregulation of p300 histone acetyltransferase activity by both genetic and pharmacological approaches induces cell senescence via a mechanism that is independent of the activation of either p53 or p16 axis [45], we can speculate that a similar independent mechanism is also activated in A-485-treated H1299 cells.

In addition, our studies demonstrate for the first time that pharmacological targeting of the p300/CBP HAT domain by A-485 induces a canonical autophagic flux. Our findings are also in accordance with a recent report showing autophagy induction upon inhibition of p300 in *C. elegans* and embryonic cells [46], supporting the hypothesis that the function of p300 on autophagy can also be pharmacologically regulated. Functional interaction between autophagy, senescence, and apoptosis in response to p300/CBP inhibition has not been demonstrated so far. In our studies, A-485 induces a canonical autophagic flux which counteracts A-485-induced apoptotic cell death and leads to cellular senescence. In particular, disruption of A-485-induced autophagy by genetic or pharmacological approaches triggered apoptotic cell death, thus indicating that autophagy acts as a bona fide cytoprotective mode to evade cell death. This idea is supported by previous studies that combine inhibitors of other epigenetic enzymes with autophagy inhibitors [47, 48], providing a conceptual framework for the development of combinatorial strategies for HAT inhibitors. Digging deeper into the mechanisms by which A-485 induces autophagy, we found increased ROS levels, a well-established inducer of several processes. It is interesting to remark that the moderate level of ROS generation may underlie the lack of massive apoptosis observed in A-485-treated NSCLC cells.

Notably, our results also show that blocking oxidative stress by NAC attenuated A-485-induced ROS production, autophagy, and senescence, confirming that ROS upregulation has a key role in the upstream events leading to autophagy and senescence commitment. We also found a time-dependent activation of stress-responsive kinases p38 and an increased level of Foxo1, a p300/CBP regulated, ROS-responsive transcription factor required for autophagy in cancer cell lines [49, 50]. In line with studies showing that the generation of ROS leads to DNA damage and senescence [51, 52], we also observed that A-485 induces nuclear accumulation of γH2AX, a marker of DNA double-strand breaks. These data suggest that A-485 triggers a DNA damage response, although it remains to be established whether ATM and ATR, two key players in the DNA damage response, are activated in response to p300/CBP inhibition.

To our knowledge, this is the first demonstration of the presence of a regulatory interplay, in which the autophagy pathway governs the cell fate choice between senescence and apoptosis in response to p300/CBP inhibition in a lung cancer setting. These findings offer the potential for further preclinical investigations using inhibitors of autophagy. Further studies will be needed to establish whether autophagy is directly required for the establishment of the senescence phenotype in response to A-485 inhibition.

In conclusion, the present study highlights the role of CBP/p300 in driving a redox/autophagy axis leading to senescence-induced growth arrest in non-small cell lung cancer cells and brings novel insight into the regulation of cellular pathways controlled by a major acetyltransferase family, i.e., p300/CBP, in lung cancer cells, involving acetylation, ROS formation, DNA damage, autophagy, and senescence activation.

## Materials and methods

### Cell cultures and reagents

Human NSCLC cells were cultured in RPMI1640 (NCI-H460, NCI-H1650, and H1299) or in DMEM high glucose medium (A549) and maintained at 37 °C in a humidified atmosphere (95% air and 5% CO_2_) supplemented with 10% fetal bovine serum, L-Glutamine and antibiotics. H1299 cells expressing shBeclin-1, sh-Control, EGFP-LC3B, or mRFP-EGFP-LC3B were generated in our previous studies [53]. All cell lines were obtained from Dr. Donatella Del Bufalo and routinely tested for mycoplasma infection by 4′,6-diamidino-2-phenylindole (DAPI, Sigma-Aldrich, Saint Louis, USA) staining. A-485 was synthetized as previously described [25], dissolved in DMSO, and stored at −20 °C. For all experiments, cells were treated with 0.1% DMSO, as a control. Chloroquine diphosphate (CQ, Sigma-Aldrich) was dissolved in water and *N*-Acetyl-l-cysteine (NAC, Sigma-Aldrich) was dissolved in the medium and used freshly.

### Cell proliferation, cell cycle, reactive oxygen species (ROS), and senescence analyses

To test the effect of A-485 on cell growth, 5 × 10^4^ cells/dish were plated in 60 mm petri-dish. After 24 h, exponentially growing cells were treated with the A-485 inhibitor at concentrations ranging from 1 to 20 μM for 24–120 h. Adherent cells were collected at every time point, and cell number was determined by using Cell Counter (Beckman Coulter, Brea USA).

To test the effect of A-485 on cell viability, a 3-(4,5-Dimethylthiazol-2-yl)-2,5-Diphenyltetrazolium Bromide (MTT) assay was performed as previously described [54].

For clonogenicity assay, treated cells were trypsinized after 72 h treatment, resuspended as single cells, and plated in 60 mm Petri-dishes with 3000 or 5000 cells per plate in triplicate. After 10 days, the colonies were fixed with methanol and stained with 1% crystal violet for 10 min.

Cell size and granularity was evaluated by analysing the side scattering (SSC-H) and forward scattering (FSC-H) of unstained cells by flow cytometer (20,000 cells per analysis).

The distribution of cells through cell cycle phases was analysed in both floating and adherent cells by flow cytometer as previously reported [55].

ROS production was analysed by using DCFDA/H_2_DCFDA - Cellular ROS Assay Kit (ab113851, Abcam Cambridge, UK). Cells were treated with hydrogen peroxide (H_2_O_2_, 10 mM, 15 min), a treatment that elicits ROS production, as a positive control. Briefly, adherent cells were collected, washed with PBS, stained with an H_2_DCFDA probe for 30 min, and quickly analyzed with a flow cytometer. At least 10,000 events were analyzed.

For senescence-associated-β-galactosidase (SA-β-Gal) assay, fixed cells were incubated in staining solution: 1 mg/ml 5-Bromo-4-chloro-3-indolyl-β-d-galactoside, 5 mM potassium ferricyanide, and 2 mM MgCl_2_ in PBS, pH 6.0. Blue cells were visualized in bright field microscopy and counted. In some senescence experiments, cells were pretreated with NAC (10 mM) for 4 h before the addition of A-485 and stained as previously described [56].

### Live cell time-lapse microscopy

In total, 3 × 10^4^ A549 cells/well were seeded in 4-well μ-slides (80426, Ibidi, Martinsried, DE). After 24 h, cells were treated with different doses of A-485 and time-lapse recording started. Cells were recorded under an Eclipse Ti inverted microscope (Nikon, Tokyo, JA), using a Plan Fluor 40x/0.6 NA objective (Nikon) for DIC; during the whole observation, cells were kept in a microscope stage incubator (Basic WJ, Okolab, Naples, IT) at 37 °C and 5% CO_2_. Images were acquired using a 40x objective for 5 days at every 7 min-interval. For every treatment condition, we plotted each recorded cell over the time axis and used a color code to represent cellular processes: timing of mitotic entry, appearance and persistence of cytoplasmic vacuoli, and apoptotic features. Videos and still images were processed using NIS-Elements AR 4.0.

### Immunofluorescence and autophagic flux analyses

Cells grown on glass coverslips were rinsed in PBS, fixed in 3.7% formaldehyde in PBS, permeabilized in 0.5% Triton X-100, and then blocked with 20% normal goat serum in PBS for 1 h at RT. Coverslips were processed for immunofluorescence using the following antibodies: Phospho-γH2AX (Ser139, Rabbit, #4353, 1:200), Tri-Methyl-Histone H3 (Lys9, Rabbit #1396, 1:400), both from Cell Signaling, Danvers MA, USA. Secondary antibodies conjugated to Alexa Fluor 488 and Cy3 were also used. DNA was counterstained with 0.05 μg/ml DAPI.

Detection of autophagic structures was obtained by fluorescence microscopy observing LC3B puncta in H1299-EGFP-LC3B expressing cells. Typically, at least 100 cells were counted, and cells with more than 10 puncta were considered autophagy positive. Preparations were examined under either an Olympus AX70 microscope, using a 40x or 100×/1.35 NA objective, or a Nikon Eclipse 90i microscope. Single-cell images were taken using an immersion oil 100x or 40x objective. Images were processed using the maximum intensity projection method and the “spot detection” and “count objects” tools of NIS-Element AR 5.02 were used. Automated quantification of H3K9me3 fluorescence intensity signals was employed: images were analyzed using open-source Cell Profiler 4.1.3 image analysis software (https://cellprofiler.org/) to measure fluorescence-integrated intensity values.

### Western blot analysis

At the end of each treatment, both floating and adherent cells were collected, lysed in RIPA lysis buffer (50 mM Tris-HCL pH 8, 150 mM NaCl, 1 mM EGTA, 0.5% Sodium deoxycholate, 1 mM EDTA, 1% NP40, 0.1% SDS) containing protease and phosphatase inhibitor cocktail (Roche, Basilea, CH). Between 30 and 50 μg of total protein were resolved under reducing conditions and protein extracts were fractionated by SDS-PAGE in 10–13.5% gel, transferred to a nitrocellulose filter using wet transfer, blocked with 5% BSA or low fat dry milk, and subjected to immunoblot assay. The following antibodies were used for immunodetection: Acetyl-H3 (Lys27, Cell Signaling #4353, 1:1000); Acetyl-H3 (Lys18, Cell Signaling #9675, 1:1000); LC3B (Sigma-Aldrich L8918, 1:1000); p62 (Santa Cruz Biotechnologies sc-28359, 1:200, Santa Cruz, USA); ATG7 (Cell Signaling #2631, 1:1000); p53 (Cell Signaling #48818, 1:1000,); Phospho-p53 (Cell Signaling, Ser 15 #9286 1:1000) Beclin-1 (Cell Signaling #3738, 1:1000); Phospho-γH2AX (Cell Signaling Ser139,#2577, 1:1000); Histone H2A (Cell Signaling #12349, Rabbit 1:1000,);p38 MAPK Antibody (Cell Signaling, #9212,1:500); Phospho-p38 MAPK (Thr180/Tyr182, Cell Signaling, #4511, 1:500) p21 (Cell Signaling #2947, 1:500); PARP (Sigma-Aldrich AB3565, 1:200); GAPDH (Santa Cruz Biotechnologies sc-32233, 1:1000). Secondary antibodies were either anti-mouse or anti-rabbit immunoglobulin G (IgG)-horseradish peroxidase conjugated antibodies (Biorad Laboratories, Hercules, USA). Membranes were again washed with TBST, and signals were detected by enhanced chemiluminescence (WESTAR ECL Substrates). A densitometric evaluation was performed using Image Lab software and values normalized to relative controls, depending on the analysis.

### Statistics

Experiments were replicated three times unless otherwise indicated, and the data were expressed as means ± standard deviation (SD) or mean ± standard error (SEM). Differences between groups were analyzed with a two-sided paired or unpaired *t*-test while one-way analysis of variance (ANOVA) was applied for multi-group data comparison, followed by an indicated test for multiple comparison. They were statistically significant for *p* < 0.05.

## Supplementary information

SUPPLEMENTAL MATERIAL

## Data Availability

The data that support the findings of this study are present in the paper and available from the corresponding author on reasonable request.
