# Supplementary material for: Pharmacological targeting of CBP/p300 drives a redox/autophagy axis leading to senescence-induced growth arrest in non-small cell lung cancer cells
Source: Cancer Gene Ther. 2022 Sep 18;30(1):124–36. doi: 10.1038/s41417-022-00524-8 (PMC9842509; doi:10.1038/s41417-022-00524-8)
Supplement: Supplementary file 1 — SUPPLEMENTAL MATERIAL [file 41417_2022_524_MOESM1_ESM.pdf]

FIGURE S1

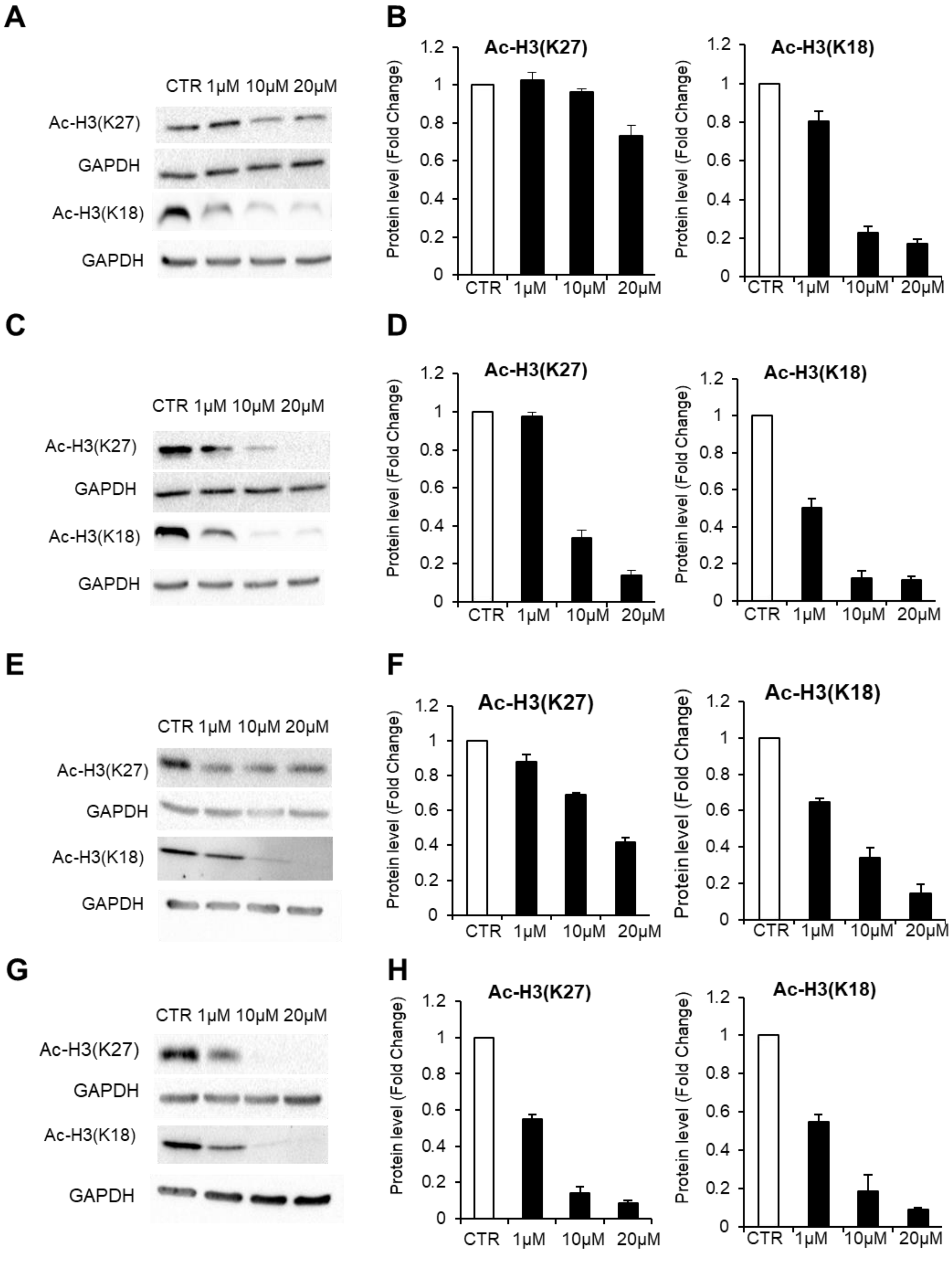
A
B
C
D
E
F
G
H
CTR 1µM 10µM 20µM
Ac-H3(K27)
GAPDH
Ac-H3(K18)
Protein level (Fold Change)
1.2
1
0.8
0.6
0.4
0.2
0
CTR
1µM
10µM
20µM

Fig 2S

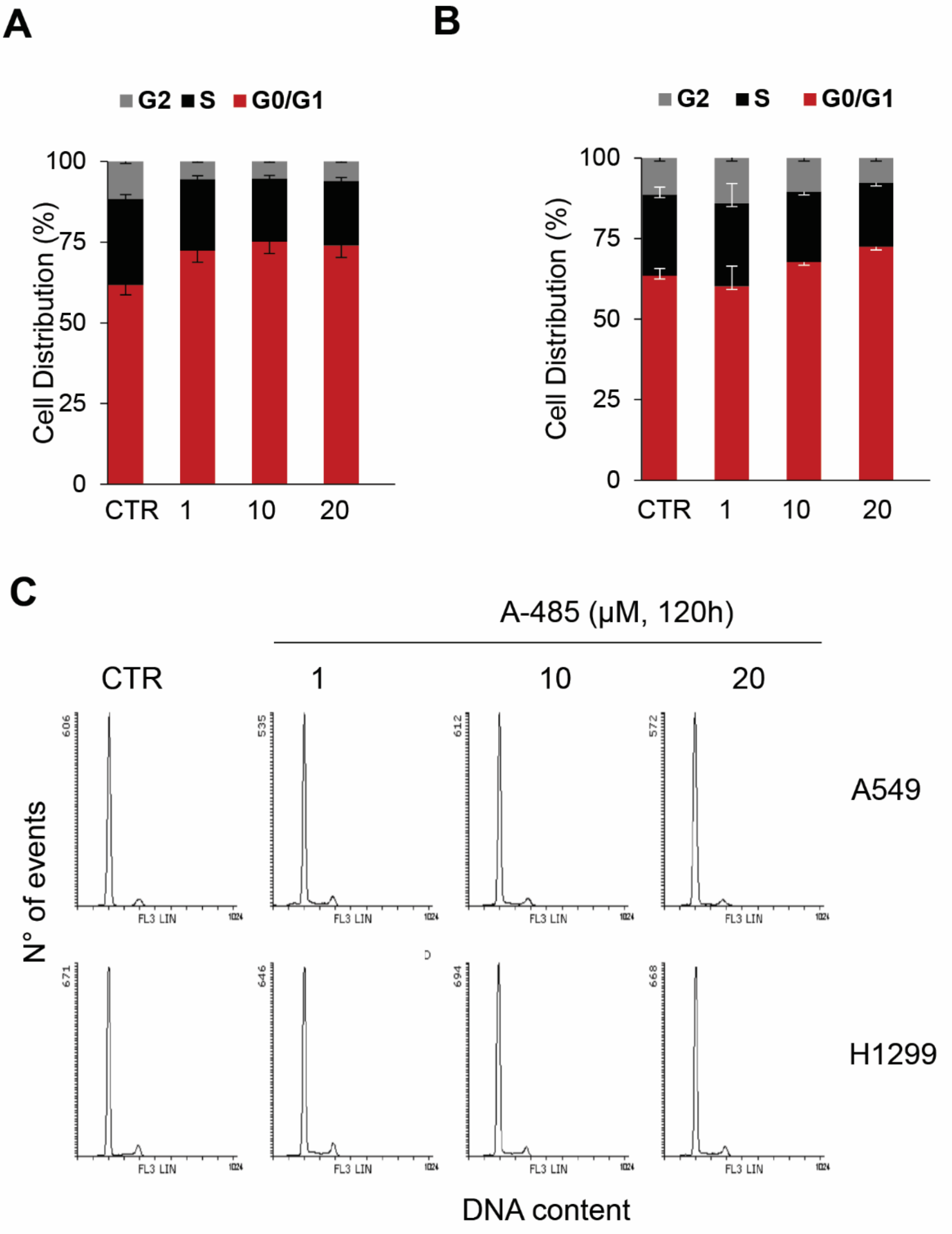
A
B
G2 S G0/G1
100
75
50
25
0
Cell Distribution (%)
CTR 1 10 20
C
A-485 (μM, 120h)
CTR
1
10
20
A549
H1299
N° of events
DNA content
FL3 LIN
606
535
612
572
671
646
694
668

SUPPLEMENTARY FIGURE LEGEND

Figure S1. A-485 reduces Histone H3 acetylation (Ac-H3) at lysine K18 and K27 in NSCLC cell lines. Western Blot analyses of histone H3 acetylated at lysine residues K18 and K27 in A549 (**A-D**) and H1299 (**E-H**) cells treated with A-485 at the indicated concentrations for 24 (**A,B,E.F**) or 48 (**C,D,G,H**) h. Western blots representative of three independent experiments with similar results are shown. **(B,D,F,H)** Ac-H3 levels were quantified by densitometric analyses using ImageLab software and the relative levels of proteins were expressed in the histograms as fold changes of treated versus untreated samples, after GAPDH normalization. Results represent mean ± SD of three independent experiments. CTR represents 0.1% DMSO treated cells.

Figure S2. A-485 causes G0/G1 arrest in NSCLC cell lines. (**A,B**) The histograms represent the percentage of A549 (**A**) and H1299 (**B**) cells in different cell cycle phases, either untreated or treated with A-485 for 72 h. **(C)** Representative flow cytometric profiles of the cell cycle distribution by propidium iodide staining in A549 and H1299 cells untreated or treated with different concentration of A-485 at 120 h. CTR represents 0.1% DMSO treated cells.
